# Molecular Therapies in Thyroid Cancer

**DOI:** 10.3390/ph4010091

**Published:** 2010-12-24

**Authors:** Timothy Huyck, Mark Agulnik

**Affiliations:** Northwestern University, Feinberg School of Medicine, Division of Hematology/Oncology, Department of Medicine, 676 North St. Clair Street, Suite 850, Chicago, IL 60611, USA

**Keywords:** advanced thyroid cancer, molecular therapy, tyrosine kinase inhibitors

## Abstract

Thyroid cancer is a common diagnosis with greater than 34,000 cases per year in the United States. Early stage thyroid cancer is often managed with surgical intervention and radioactive iodine; however, for recurrent or metastatic disease, the treatment options, historically, have been limited to chemotherapy. Chemotherapy for metastatic thyroid cancer has been of limited efficacy. Encouragingly, molecular therapeutics have played a greater role in managing patients with advanced disease. These agents work primarily through disruption of tyrosine kinase pathways. This review will discuss the expanding role of molecular targets in managing patients with advanced thyroid cancer.

## 1. Introduction

Worldwide, thyroid cancer is the 17^th^ most common cancer, with over 140,000 new cases per year. More than 75% of newly diagnosed cases are seen in the female population. Thyroid cancer has an estimated worldwide annual mortality rate of greater than 35,000 [1]. In the United States (U.S.), the incidence and mortality rate of thyroid cancer has been increasing. Reasons for this increase are not clear, but are felt to be related to better diagnosis and changing pathologic criteria [2]. In 2009, the annual incidence of thyroid carcinoma in the U.S. was 34,000. In that same time period the annual cancer related deaths experienced by persons living with thyroid cancer was greater than 1,600 [3]. In the U.S. population the lifetime risk of developing thyroid cancer is less than 1% (0.83% in women and 0.33% in men) [4]. The only known environmental risk factor for the development of thyroid cancer is exposure to ionizing radiation a risk that appears increased in children and those patients with a family history of thyroid carcinoma [5].

Thyroid carcinoma is usually asymptomatic at time of diagnosis with 50% of malignant nodules discovered during routine physical exam, on imaging obtained for other reasons, or during surgery for benign disease. The other 50% are initially appreciated by the patient as an asymptomatic nodule [6] Metastatic disease often will present on imaging or with symptoms related to the organ involved. Common sites of metastases include the lung, bone or the central nervous system [7-9].

There are three main histologic types of thyroid cancer: differentiated, medullary and anaplastic. Differentiated thyroid cancers, including papillary, follicular and Hurthle subtypes, account for 90% of all thyroid malignancies [10]. The majority of these tumors are curable with primary therapy which consists of surgery when feasible, followed in many patients by radioiodine and thyroxine therapy [11]. Medullary thyroid cancer originates from parafollicular cells and accounts for 3-5% on newly diagnosed cases per year, but a disproportionate degree of mortality [12]. Anaplastic thyroid cancer, similar to the differentiated subtypes, originates from thyrocytes but accounts for less than 2% of all thyroid cancers [10]. It is almost universally fatal as it is not sensitive to raidioindine and is often not amenable to surgery because of early metastases and/or extensive local invasion.

Patients with advanced thyroid cancer are frequently offered surgery or radioiodine therapy, if the tumor is still iodine avid, under either a curative or palliative intent. External beam radiation therapy is utilized for symptomatic metastases. Once thyroid cancer metastasizes to distant sites and loses its ability to concentrate iodine, it is not amenable to radioactive iodine or surgery, the expected survival declines rapidly [8,13]. Chemotherapy is the only viable option for systemic management but has been uniformly disappointing [14]. Traditional cytotoxic chemotherapeutic agents such as doxorubicin and cisplatin are generally associated with no more than 25% partial response rates, complete remission is rare and toxicities from these treatments are considerable [15]. Doxorubicin monotherapy, which remains the only treatment of metastatic thyroid cancer approved by the U.S. Food and Drug Administration, is occasionally effective when dosed appropriately (60-75 mg/m^2^ every 3 weeks), but durable responses are uncommon [16-18]. The combination of additional therapy to doxorubicin has not proven to be of significant benefit [19-21]. Due to the limited efficacy of chemotherapy in advanced thyroid cancer and given certain characteristics associated with the biology seen frequently in thyroid cancer, molecular therapies are being investigated as an option in management.

## 2. Molecular Therapies

Molecular agents, focused on inhibition of tyrosine kinase pathways, are expanding treatment options for patients with a broad spectrum of tumors, including thyroid cancer. Molecular agents are directed at proteins expressed on the surface of cells or at products of protein binding. The molecular targets include vascular endothelial growth factor inhibitors (VEGF), epidermal growth factor inhibitors (EGFR), and mammalian target of rapamycin (mTOR) inhibitors.

Angiogenesis is the formation and maintenance of new blood vessels that enable survival, growth, and metastases of tumor cells. Kinases function as signaling intermediates that stimulate tumor proliferation, angiogenesis, invasion, metastasis, and avoidance of apoptosis. Most cancers, thyroid and otherwise, eventually acquire autonomy from native, healthy tissue and are able to activate these kinase proteins [22]. These proteins have been isolated and include vascular endothelial growth factor (VEGF) which stimulates angiogenesis by attaching to VEGF receptors on endothelial cells [23,24]. These new vessels allow for tumor propagation and metastases. Inhibition of these proteins and similar molecules downstream from VEGF has proven, in early phase trials, to be efficacious in management of thyroid cancer.

### 2.1. Aflibercept

Aflibercept is a VEGF-Trap (VT) compound which is recombinantly produced fusion protein consisting of human sequences for VEGF receptor extracellular domains and human immunoglobulin which effectively suppresses tumor growth. A phase II trial was initiated utilizing aflibercept in radioactive iodine refractory thyroid cancer. Aflibercept 4 mg/kg IV was dosed on a two week cycle until discontinued due to disease progression or tolerability. No complete or partial responses were appreciated, 18 patients had stable disease and the median duration of stable disease was 178 days. Side effects included proteinuria, lymphopenia and reversible encephalopathy [25].

### 2.2. Axitinib

A common finding in thyroid cancer of all histologic subtypes is the increased levels of VEGF compared to a healthy thyroid organ [26-28]. Multiple pre-clinical studies have shown that axitinib rapidly and selectively inhibits VEGF 1, 2, and 3 activity and thus resultant angiogenesis in tumor models [29-32] Axitinib is an potent, oral, and selective inhibitor of VEGF receptors 1, 2, and 3. Cohen and colleagues [33] enrolled 60 patients with advanced, incurable thyroid cancer not amenable to surgery or radioiodine therapy. Patients were enrolled onto a single phase II trial to receive axitinib 5 mg orally twice daily, which was dose escalated to 7 mg orally twice daily and again to 10 mg orally twice daily, as patients tolerated therapy with no grade 2 or greater adverse events. Partial response rates were observed in 18 patients for an overall response rate of 30%. Stable disease lasting > or = to 16 weeks was seen in 23 patients. Responses were seen in patients of all histologic subtypes. Median progression free survival was 18.1 months. Treatment related adverse events that were grade 3 or greater included hypertension, fatigue, diarrhea, weight loss, headache and proteinuria.

### 2.3. Bortezomib

Bortezomib is a reversible proteasome inhibitor. Inhibition of the proteasome pathway affects multiple signaling pathways including the RAS/RAF pathway leading to cell cycle arrest and apoptosis. In a phase I clinical trial bortezomib was used in combination with sunitinib in patients with radioiodine-refractory thyroid cancer. Seven patients were enrolled in the trial. Two patients (33%) had a partial response and four patients (66%) had stable disease, one patient was not evaluable at the time of publication. Bortezomib was given in a dose escalating fashion (1-1.6 mg/m^2^ weekly) and standard doses of sunitinib (37.5 mg or 50 mg daily) were used. There were no dose limiting toxicities but thrombocytopenia, neutropenia and hypertension were appreciated [34]. Bortezomib is FDA approved for therapy in multiple myeloma and mantle cell lymphoma.

### 2.4. Everolimus

Everolimus is a mTOR inhibitor which is currently being evaluated for use when patients have progression of disease on sorafenib. Thirty-five patients with iodine-resistant differentiated thyroid cancer are being enrolled in a phase II trial at time of progression on sorafenib. Patients are continued on sorafenib 200 mg po BID and in addition are given everolimus 5 mg po daily. Doses will be escalated as toxicities allow to a maximum of 400 mg BID of sorafenib and 10 PO daily of everolimus. This trial continues to enroll and results are pending [35].

### 2.5. Gefitinib

A tyrosine kinase inhibitor of the EGFR, gefitnib has been studied in non-small cell cancer [36], and due to an up regulation of EGFR on normal and malignant thyroid tissue phase II trials have been undertaken. In one study of 27 patients there were no partial or complete responses, but eight patients had tumor reduction that did not qualify as a partial response and one patient with anaplastic histology had stable disease for 12 months [37]. Rash, weight loss, nausea and oral ulcers are frequent side effects associated with gefitinib. Currently gefitinib is FDA approved for locally advanced or metastatic non-small cell cancer of the lung which expresses EGFR.

### 2.6. Motesanib

Another multi-kinase agent, motesanib is an oral inhibitor of the tyrosine kinases of VEGF receptors 1, 2 and 3, platelet-derived growth factor (PDGF) and KIT (a downstream target of VEGF) [38]. An early phase I study in patients with advanced solid tumors found motesanib to be well tolerated and have evidence of antitumor activity [39]. Sherman and colleagues treated 93 patients in a phase 2 study with progressive, locally advanced or metastatic, radioiodine-resistant differentiated thyroid cancer with motesanib. Patients were initiated on motesanib 125 mg orally once daily and monitored for progression of disease and side effects of therapy. Partial response was appreciated in 13 patients with 62 having stable disease. Median progression free survival was estimated at 40 weeks [40]. Schlumberger et al performed a phase II trial which evaluated motesanib in patients with locally advanced, metastatic, or symptomatic MTC. Patietnts were given motesanib 125 mg orally daily for up to 48 weeks or until unacceptable toxicity or disease progression. Ninety one patients were enrolled, two achieved objective response, 74 patients had stable disease with 48% of these patient having durable stable disease, defined as greater than or equal to 24 weeks [41]. Common side effects of motesanib therapy included diarrhea, hypertension, fatigue, hypothyroidism and anorexia. Further trials need to be studied to evaluate the efficacy of motesanib among all histologic subtypes of thyroid cancer.

### 2.7. Pazopanib

A tyrosine kinase inhibitor targeting VEGF receptors, PDGF and C-KIT, pazopanib was assessed in a phase II trial as an active agent in metastatic radioiodine-refractory differentiated thyroid cancer. The study enrolled 39 patients. Each patient received pazopanib 800 mg po daily in 4- week cycles until disease progression, drug intolerance or both. Clinical outcomes were assessed in 37 patients and partial responses were appreciated in 18, a response rate of 49%. Dose reductions were necessary in 16 patients with the most frequent side effects including fatigue, skin and hair hypopigmentation, diarrhea and nausea [42]. Further studies are underway further evaluating this agent in patients with differentiated thyroid cancer, as well as in patients with medullary and anaplastic thyroid cancer.

### 2.8. Sorafenib

Sorafenib is an oral, multitargeted tyrosine kinase inhibitor with activity against VEGF receptors 2 and 3. It also shows inhibition of RET oncogene and the serine kinase BRAF, a downstream target in the VEGF pathway. Given the predisposition of RET activity particularly in medullary thyroid cancer, the anit-RET activity of sorafenib was felt to make this drug an active agent against thyroid cancer [43]. Multiple early phase clinical trials have found sorafenib to be a well tolerated option with antitumor activity in metastatic iodine-refractory thyroid cancer. In one phase II trial 30 patients were enrolled and initiated on sorafenib 400 mg po twice daily. Seven patients had a partial response lasting 18–84 weeks, 16 patients had stable disease lasting 14-89 weeks, with the median progression free survival being 79 weeks [44]. A second phase II trial of sorafenib targeting RAF and VEGF receptor kinases in papillary thyroid cancer (PTC) was also undertaken. This trail had two arms, arm A assessed patients with chemotherapy-naïve PTC. Arm B patients had other subtypes of metastatic thyroid cancer or previous chemotherapy. Patient were given sorafenib 400 mg orally twice daily, with responses assessed every two months with the primary endpoint being objective response rate based on RECIST criteris. Of the 41 patients evaluated, six patients had a partial response, 23 patients had stable disease longer than six months. Median progression free survival was 15 months [45]. Common toxicities of sorafenib include desquamating rash of the hands and feet, and gastrointestinal symptoms including nausea, diarrhea and weight loss. Sorafenib is approved by the U.S. FDA for treatment of renal cell cancer and hepatocellular carcinoma, its multitargeted kinase inhibition makes it an attractive agent for many tumors and its role in thyroid cancer is being further evaluated in ongoing phase III clinical trials.

### 2.9. Sunitinib

An oral, small-molecule tyrosine kinase inhibitor of VEGF 1, 2 and 3, and RET, sunitinib was initially looked at in a small series of patients who saw a partial response receiving 50 mg orally daily times 28 days followed by 14 days with no therapy [46]. Preliminary results, from Cohen and colleagues, of a phase II trial in patients with progressive differentiated thyroid cancer or medullary thyroid cancer reports a partial response rate of 13% in 31 differentiated thyroid cancer patients. There were no partial responders in those patients who had medullary cacner. Stable disease was seen in 68% of differentiated thyroid cancer and 83% of medullary thyroid cancer patients [47]. In more recent studies, two complete responses and eight partial responses were reported in a phase II trial of 29 patients with either metastatic medullary or differentiated thyroid cancer whose metastatses were PET avid. This patient cohort received 37.5 mg orally continuously until either disease progression or side effects which mandated discontinuation of the medication [48]. Hypertension and resultant cardiomyopathy is the most significant side effect of sunitinib. Currently sunitinib is FDA approved for us in gastrointestinal stromal tumors (GIST) and renal cell cancer.

### 2.10. Tipifarnib

Tipifarnib inhibits farnesyl transferase, preventing activation of RAS and selectively killing RAS-transformed cells. In a phase I trial, tipifarnib was used in combination with sorafenib in 35 patients with either differentiated or medullary thyroid cancer. Durable responses which was defined as a PR or SD greater than or equal to 6 months were seen in 13/15 (87%) of differentiated thyroid cancer and 9/10 (90%) of medullary thyroid cancer. Ten patients were discontinued from the study prior to the first re-staging due to toxicities which were primarily related to hand-foot skin reaction. The maximum tolerated dose of tipifarnib was 200 mg daily in split doses and 600 mg daily of sorafenib [49].

### 2.11. Vandetanib

After demonstrating potent inhibition of VEGF-2 tyrosine kinase as well as activity against VEGF 3, epidermal growth factor receptor (EGFR), and RET kinases [50], vandetanib was studied in 30 patients with metastatic familial form of medullary thyroid cancer. This phase II trial started patient on vandetanib 300 mg orally daily. A partial response was seen in 33% of those patients, and stable disease was seen in 63%. Toxicities were manageable with most common adverse events being diarrhea, rash, and asymptomatic QTC prolongation [51]. Based on the results of these and other early phase trials, Wells et al conducted a randomized, double blinded phase III trial on patients with unresectable, measurable, locally advanced or metastatic MTC. Patients were randomized 2:1 to receive vandetanib versus placebo, the primary end point was progression free survival. The data for this trial is still being accumulated but at median follow up at 24 months statistically significant PFS prolongation (HR 0.45, 95% CI 0.3–0.69, p = 0.0001) was observed [52]. Vandetanib has been submitted to the US FDA for approval in the setting of advanced medullary thyroid cancer.

## 3. Summary

Thyroid cancer is a disease which has limited treatment options when patients present with metastatic or radioiodine resistant tumors. Systemic chemotherapy has limited efficacy and a significant side effect profile. External beam radiotherapy is used only in the setting of a solitary metastatic focus or palliation of symptoms. Despite this, treatment options for patients with metastatic radioiodine resistant thyroid cancer are increasing. Advances in molecular oncology and the development of novel agents inhibiting angiogenesis, specifically VEGF receptors, have shown efficacy. Improvement in progression free survival suggests potential significant clinical benefit from therapy. Unfortunately, these novel agents have not been found to improve overall survival for patients with metastatic thyroid cancer. The toxicity profile of many of the tyrosine kinase inhibitors, while less than cytotoxic chemotherapy, can be dose limiting and significant. The low rate of partial response and the clinician's inability to predict who will and who will not respond to therapy dictate that further clinical trials, both with newer more effective agents and in combination with systemic therapy, are needed.

## References

[1] Parkin D.M., Bray F., Ferlay J., Pisani P. (2005). Global cancer statistics, 2002. CA Cancer J. Clin..

[2] Davies L., Welch H.G. (2006). Increasing incidence of thyroid cancer in the United States, 1973-2002. JAMA.

[3] Jemal A., Siegel R., Ward E., Hao Y., Xu J., Thun M.J. (2009). Cancer statistics, 2009. CA Cancer J. Clin..

[4] (2009). SEER Cancer Statistics Review, 1975-2006. http://seer.cancer.gov.csr/1975_2006/results_merged/sect_26_thyroid.pdf.

[5] Wong F.L., Ron E., Gierlowski T., Schneider A.B. (1996). Benign thyroid tumors: general risk factors and their effects on radiation risk estimation. Am. J. Epidemiol..

[6] Kaplan M. (2005). Clinical Evaluation and Management of Solitary Thyriod Nodules.

[7] Mazzaferri E.L., Jhiang S.M. (1994). Long-term impact of initial surgical and medical therapy on papillary and follicular thyroid cancer. Am. J. Med..

[8] Ruegemer J.J., Hay I.D., Bergstralh E.J., Ryan J.J., Offord K.P., Gorman C.A. (1988). Distant metastases in differentiated thyroid carcinoma: a multivariate analysis of prognostic variables. J. Clin. Endocrinol. Metab..

[9] Samaan N.A., Schultz P.N., Haynie T.P., Ordonez N.G. (1985). Pulmonary metastasis of differentiated thyroid carcinoma: treatment results in 101 patients. J. Clin. Endocrinol. Metab..

[10] Green L.D., Mack L., Pasieka J.L. (2006). Anaplastic thyroid cancer and primary thyroid lymphoma: a review of these rare thyroid malignancies. J. Surg. Oncol..

[11] Sherman S.I. (2003). Thyroid carcinoma. Lancet.

[12] Schlumberger M., Carlomagno F., Baudin E., Bidart J.M., Santoro M. (2008). New therapeutic approaches to treat medullary thyroid carcinoma. Nat. Clin. Pract. Endocrinol. Metab..

[13] Shoup M., Stojadinovic A., Nissan A., Ghossein R.A., Freedman S., Bennnan M.F., Shah J.P., Shaha A.R. (2003). Prognostic indicators of outcomes in patients with distant metastases from differentiated thyroid carcinoma. J. Am. Coll. Surg..

[14] Ain K.B. (1995). Papillary thyroid carcinoma. Etiology, assessment, and therapy. Endocrinol. Metab. Clin. North Am..

[15] Sarlis N.J. (2001). Metastatic thyroid cancer unresponsive to conventional therapies: novel management approaches through translational clinical research. Curr. Drug Targets Immune. Endocr. Metabol. Disord..

[16] Gottlieb J.A., Hill C.S. (1974). Chemotherapy of thyroid cancer with adriamycin. Experience with 30 patients. N. Engl. J. Med..

[17] Gottlieb J.A., Hill C.S., Ibanez M.L., Clark R.L. (1972). Chemotherapy of thyroid cancer. An evaluation of experience with 37 patients. Cancer.

[18] O'Bryan R.M., Baker L.H., Gottlieb J.E., Rivkin S.E., Balcerzak S.P., Grumet G.N., Salmon S.E., Moon T.E., Hoogstraten B. (1977). Dose response evaluation of adriamycin in human neoplasia. Cancer.

[19] Argiris A., Agarwala S.S., Karamouzis M.V., Burmeister L.A., Carty S.E. (2008). A phase II trial of doxorubicin and interferon alpha 2b in advanced, non-medullary thyroid cancer. Invest. New Drugs.

[20] Casara D., Rubello D., Saladini G., Masarotto G., Facero A., Girelli M.E., Busnardo B. (1993). Different features of pulmonary metastases in differentiated thyroid cancer: natural history and multivariate statistical analysis of prognostic variables. J. Nucl. Med..

[21] Scherubl H., Raue F., Ziegler R. (1990). Combination chemotherapy of advanced medullary and differentiated thyroid cancer. Phase II study. J. Cancer Res. Clin. Oncol..

[22] Hanahan D., Weinberg R.A. (2000). The hallmarks of cancer. Cell.

[23] Folkman J. (1971). Tumor angiogenesis: therapeutic implications. N. Engl. J. Med..

[24] Sivakumar B., Harry L.E., Paleolog E.M. (2004). Modulating angiogenesis: more *vs*. less. JAMA.

[25] Sherman E. (2010). A phase II study of VEGF trap (aflibercept) in patients with radioactive iodine-refractory, positron emission tomography (PET) positive thyroid carcinoma. J. Clin. Oncol..

[26] Bauer A.J., Patel A., Terrell R. (2003). Systemic administration of vascular endothelial growth factor monoclonal antibody reduces the growth of papillary thyroid carcinoma in a nude mouse model. Ann. Clin. Lab. Sci..

[27] Schoenberger J., Grimm D., Kossmehl P., Infanger M., Kurth E., Eilles C. (2004). Effects of PTK787/ZK222584, a tyrosine kinase inhibitor, on the growth of a poorly differentiated thyroid carcinoma: an animal study. Endocrinology.

[28] Viglietto G., Maglione D., Rambaldi M. (1995). Upregulation of vascular endothelial growth factor (VEGF) and downregulation of placenta growth factor (PlGF) associated with malignancy in human thyroid tumors and cell lines. Oncogene.

[29] Baffert F., Le T., Sennino B., Thurston G., Kuo C.J., Hu-Lowe D., McDonald D.M. (2006). Cellular changes in normal blood capillaries undergoing regression after inhibition of VEGF signaling. Am. J. Physiol. Heart Circ. Physiol..

[30] Inai T., Mancuso M., Hashizume H., Baffert F., Haskell A., Baluk P., Hu-Lowe D.D., Shalinsky D.R., Thurston G., Yancopoulos G.D., McDonald D.M. (2004). Inhibition of vascular endothelial growth factor (VEGF) signaling in cancer causes loss of endothelial fenestrations, regression of tumor vessels, and appearance of basement membrane ghosts. Am. J. Pathol..

[31] Kamba T., Tam B.Y., Hashizume H., Haskell A., Sennino B., Mancuso M.R., Norberg S.M., O'Briend S.M., Davis R.B., Gowen L.C., Anderson K.D., Thurston G., Joho S., Springer M.L., Kuo C.J., McDonald D.M. (2006). VEGF-dependent plasticity of fenestrated capillaries in the normal adult microvasculature. Am. J. Physiol. Heart Circ. Physiol..

[32] Mancuso M.R., Davis R., Norberg S.M., O'Brien S., Sennino B., Nakahara T., Yao V.J., Inai T., Brooks P., Freimark B., Shalinsky D.R., Hu-Lowe D.D., McDonald D.M. (2006). Rapid vascular regrowth in tumors after reversal of VEGF inhibition. J. Clin. Invest..

[33] Cohen E.E., Rosen L.S., Vokes E.E., Kies M.S., Forastiere A.A., Worden F.P., Kane M.A., Sherman E., Kim S., Bycott P., Tortorici M., Shalinsky D.R., Liau K.F., Cohen R.B. (2008). Axitinib is an active treatment for all histologic subtypes of advanced thyroid cancer: results from a phase II study. J. Clin. Oncol..

[34] Harvey H. (2010). Combination therapy with sunitinib and bortezomib in adult patients with radioiodine refractory thyroid cancer. J. Clin. Oncol..

[35] Brose M.S., Troxel A.B., Mamtani R. (2010). Phase II trial of everolimus with sorafenib for patients with differentiated thyroid cancer who progress on sorafenib alone. J. Clin. Onco.

[36] Lynch T.J, Bell D.W., Sordella R., Gurubhagavatula S., Okimoto R.A., Brannigan B.W., Harris P.L., Haserlat S.M., Supko J.G., Haluska F.G., Louis D.N., Christiani D.N., Settleman J., Haber D.A. (2004). Activating mutations in the epidermal growth factor receptor underlying responsiveness of non-small-cell lung cancer to gefitinib. N. Engl. J. Med..

[37] Pennell N.A., Daniels G.H., Haddad R.I., Ross D.S., Evans T., Wirth L.J., Fidias P.H., Temel J.S., Gurubhagavatula S., Heist R.S., Clark J.R., Lynch T.J. (2008). A phase II study of gefitinib in patients with advanced thyroid cancer. Thyroid.

[38] Polverino A., Coxon A., Starnes C., Diaz Z., DeMelfi T., Wang L., Bready J., Estrada J., Cattley R., Kaufman S., Chen D., Gan Y., Kumar G., Meyer J., Neervannan S., Alva G., Talvenheimo J., Montestruque S., Tasker A., Patel V., Radinsky R., Kendall R. (2006). AMG 706, an oral, multikinase inhibitor that selectively targets vascular endothelial growth factor, platelet-derived growth factor, and kit receptors, potently inhibits angiogenesis and induces regression in tumor xenografts. Cancer Res..

[39] Rosen L.S., Kurzrock R., Mulay M., Van Vugt A., Purdom M., Ng C., Silverman J., Koutsoukos A., Sun Y.N., Bass M.B., Xu R.Y., Polverino A., Wiezorek J.S., Chang D.D., Benjamin R., Herbst R.S. (2007). Safety, pharmacokinetics, and efficacy of AMG 706, an oral multikinase inhibitor, in patients with advanced solid tumors. J. Clin. Oncol..

[40] Sherman S.I., Wirth L.J., Droz J.P., Hofmann M., Bastholt L., Martins R.G., Licitra L., Eschenberg M.J., Sun Y.N., Juan T., Stepan D.E., Schlumberger M.J. (2008). Motesanib diphosphate in progressive differentiated thyroid cancer. N. Engl. J. Med..

[41] Schlumberger M.J., Elisei R., Bastholt L., Wirth L.J., Martins R.G., Locati L.D., Jarzab B., Pacini F., Daumerie C., Droz J.P., Eschenberg M.J., Sun Y.N., Juan T., Stepan D.E., Sherman S.I. (2009). Phase II study of safety and efficacy of motesanib in patients with progressive or symptomatic, advanced or metastatic medullary thyroid cancer. J. Clin. Oncol..

[42] Bible K.C., Suman V.J., Molina J.R., Smallridge R.C., Maples W.J., Menefee M.E., Rubin J., Sideras K., Morris J.C., McIver B., Burton J.K., Webster K.P., Bieber C., Traynor A.M., Flynn P.J., Goh B.C., Tang H., Ivy S.P., Erlichman C. (2010). Efficacy of pazopanib in progressive, radioiodine-refractory, metastatic differentiated thyroid cancers: results of a phase 2 consortium study. Lancet Oncol..

[43] Ball D.W. (2007). Medullary thyroid cancer: therapeutic targets and molecular markers. Curr. Opin. Oncol..

[44] Gupta-Abramson V., Troxel A.B., Nellore A., Puttaswamy K., Redlinger M., Ransone K., Mandel S.J., Flaherty K.T., Loevner L.A., O'Dwyer P.J., Brose M.S. (2008). Phase II trial of sorafenib in advanced thyroid cancer. J. Clin. Oncol..

[45] Kloos R.T., Ringel M.D., Knopp M.V., Hall N.C., King M., Stevens R., Liang J., Wakely P.E., Vasko V.V., Saji M., Rittenberry J., Wei L., Arbogast D., Collamore M., Wright J.J., Grever M., Shah M.H. (2009). Phase II trial of sorafenib in metastatic thyroid cancer. J. Clin. Oncol..

[46] Dawson S.J., Conus N.M., Toner G.C., Raleigh J.M., Hicks R.J., McArthur G., Rischin D. (2008). Sustained clinical responses to tyrosine kinase inhibitor sunitinib in thyroid carcinoma. Anticancer Drugs.

[47] Cohe E.E., Needles B.M., Cullen K.J. (2008). Phase 2 study of sunitinib in refractory thyroid cancer. J. Clin. Oncol..

[48] Carr L., Goulart B., Martins R. (2009). Phase II trial on continous dosing sunitinib in advanced, FDG-PET avid, medullary thyroid carcinoma (MTC) and well-differentiated thyroid cancer (WDTC). J. Clin. Oncol..

[49] Cabanillas M. (2010). Phase I trial of combination sorafenib and tipifarnib: The experience in advanced differentiated thyroid cancer (DTC) and medullary thyroid cancer (MTC). J. Clin. Oncol..

[50] Wedge S.R., Ogilvie D.J., Dukes M., Kendrew J., Chester R., Jackson J.A., Boffey S.J., Valentine P.J., Curwen J.O., Musgrove H.L., Graham G.A., Hughes G.D., Thomas A.P., Stokes E.S., Curry B., Richmond G.H., Wadsworth P.F., Bigley A.L., Hennequin L.F. (2002). ZD6474 inhibits vascular endothelial growth factor signaling, angiogenesis, and tumor growth following oral administration. Cancer Res..

[51] Wells S.A., Gosnell J.E., Gagel R.F., Moley J., Pfister D., Sosa J.A., Skinner M., Krebs A., Vasselli J., Schlumberger M. (2007). Vandetanib for the treatment of patients with locally advanced or metastatic hereditary medullary thyroid cancer. J. Clin. Oncol..

[52] Wells S.A. (2010). Vandetanib (VAN) in locally advanced or metastatic medullary thyrid cancer (MTC): A randomized, double-blind phase III trial (ZETA). J. Clin. Oncol..

